# 17-AAG suppresses growth and invasion of lung adenocarcinoma cells *via* regulation of the LATS1/YAP pathway

**DOI:** 10.1111/jcmm.12469

**Published:** 2015-02-25

**Authors:** Xiang-Yun Ye, Qing-Quan Luo, Yun-Hua Xu, Nai-Wang Tang, Xiao-Min Niu, Zi-Ming Li, Sheng-Ping Shen, Shun Lu, Zhi-Wei Chen

**Affiliations:** Department of Shanghai Lung Tumor Clinical Medical Centre, Shanghai Chest Hospital, Shanghai Jiao Tong UniversityShanghai, China

**Keywords:** 17-AAG, lung adenocarcinoma, growth, invasion, LATS1

## Abstract

The large tumour suppressor 1 (LATS1) signalling network has been proved to be an essential regulator within the cell, participating in multiple cellular phenotypes. However, it is unclear concerning the clinical significance of LATS1 and the regulatory mechanisms of 17-Allylamino-17- demethoxygeldanamycin (17-AAG) in lung adenocarcinoma (LAC). The aim of the present study was to investigate the correlation of LATS1 and yes-associated protein (YAP) expression with clinicopathological characteristics in LAC patients, and the effects of 17-AAG on biological behaviours of LAC cells. Subcutaneous LAC tumour models were further established to observe the tumour growth in nude mice. The results showed that the positive expression of LATS1 was significantly lowered (26.7% *versus* 68.0%, *P* < 0.001), while that of YAP was elevated (76.0% *versus* 56.0%, *P* + 0.03) in LAC tissues compared to the adjacent non-cancerous tissues; LAST1 expression was negatively correlated with YAP expression (*r* + 0.432, *P* < 0.001) and lymphatic invasion of the tumour (*P* + 0.015). In addition, 17-AAG inhibited proliferation and invasion, and induced cell apoptosis and cycle arrest in LAC cells together with increased expression of E-cadherin and p-LATS1, and decreased expression of YAP and connective tissue growth factor. Tumour volumes and weight were much smaller in 17-AAG-treated groups than those in untreated group (*P* < 0.01). Taken together, our findings indicate that decreased expression of LATS1 is associated with lymphatic invasion of LAC, and 17-AAG suppresses growth and invasion of LAC cells *via* regulation of the LATS1/YAP pathway *in vitro* and *in vivo*, suggesting that we may provide a promising therapeutic strategy for the treatment of human LAC.

## Introduction

Heat shock proteins (HSPs) are highly conserved proteins and their expression is dependent on the level of various cellular stresses. Their function is essential for normal cell viability and growth. HSP90 interacts with proteins mediating cell signalling involved in essential processes such as proliferation, cell cycle control, angiogenesis and apoptosis 1. Lung cancer tissues exhibit high expression level of HSP90, which is significantly associated with the pathological grade and lymphatic invasion of lung cancer 2, but low HSP90 expression is related with long overall survival in non-small cell lung cancer 3. HSP90 inhibition induces premature senescence in small cell lung cancer cells, and HSP90 is identified as a potential therapeutic target for lung cancer 4, indicating that intervention of HSP90 may represent a promising strategy for treatment of cancer.

Since the discovery of HSP90 as a target for anticancer therapy, the tremendous progress has been made to develop a multitude of potent HSP90 inhibitors. A promising activity is reported regarding 17-AAG in HER2-positive breast cancer and ALK-mutated lung cancers 5. 17-AAG induces the down-regulation of critical HSP90 protein clients and results in cell cycle arrest and cell apoptosis in urinary bladder cancer cells 6. Blockade of HSP90 by 17AAG antagonizes murine double minute X and synergizes with Nutlin to induce p53-mediated apoptosis in solid tumours 7. More importantly, 17-AAG as a HSP90 inhibitor can improve the therapeutic outcome of cisplatin-based combination chemotherapy against advanced bladder cancer 8, and provide new approaches to prevent and treat castration-resistant prostate cancer 9, suggesting that 17-AAG may exert inhibitory effects on tumour development. But, how the signalling pathways and factors medicate the effects of 17-AAG on cancer cells need be understood.

Furthermore, accumulating evidence indicates large tumour suppressor 1 (LATS1) as a new resident governor of cellular homeostasis. Loss of LATS1 leads to a variety of tumour types including soft tissue sarcomas, leukaemia, as well as breast, prostate, lung and oesophageal cancers 10. LATS1 as a tumour suppressor plays an important role in the control of tumour development and tumourigenesis by negatively regulating cell proliferation and modulating cell survival 11. H-warts/LATS1, a human homolog of the Drosophila warts tumour suppressor gene, which is lost in sarcomas, is of pathological importance in human sarcomagenesis 12. Recent studies establish that Hippo/LATS/yes-associated protein (YAP) pathway is one major conserved mechanism governing cell contact inhibition, organ size control and cancer development 13, and LATS1/2 can function as the tumour suppressor genes in some cancers 14–16. Moreover, some studies show that E-cadherin is lowly expressed in human lung adenocarcinoma (LAC) 17–19, and directly modulates the Hippo signalling pathway in controlling cell proliferation 14,20.

Few studies indicate the relationship between 17-AAG and LATS1 in cancers. It is found that LATS1/2 may be the HSP90 clients in several tumour cell lines including LAC A549, and 17-AAG contributes to the depletion and reduced LATS1/2 levels, leading to decreased phosphorylation of YAP and enhanced expression of connective tissue growth factor (CTGF) 21. However, the correlation of LATS1 expression with the clinicopathological characteristics of LAC patients, as well as the novel molecular mechanisms of 17-AAG in human LAC is still unintelligible. Thus, in the present study, we examined the expression of LATS1 and YAP in LAC tissues and investigated the effects and underlying mechanisms of 17-AAG on growth and invasion of LAC cells *in vitro* and *in vivo*. We hypothesized that LATS1 expression was closely correlated with tumour invasion of LAC, and 17-AAG as a HSP90 inhibitor could impair growth and invasion of LAC cells *via* regulation of the LATS1/YAP signalling.

## Materials and methods

### Materials

Human LAC tissues and corresponding adjacent non-cancerous tissues (ANCT) were from Department of Shanghai Lung Tumor Clinical Medical Centre of Shanghai Chest Hospital. LAC tissue microarray was made by Shanghai Outdo Biotech Co. Ltd (Shanghai, China). LAC A549 and LETPα-2 cell lines used in the experiments were from Institute of Biochemistry and Cell Biology (Shanghai, China). Lentivirus-mediated LATS1 vector (Lv-LATS1), Lv-LATS1-siRNA, negative control (NC) vector and virion-packaging elements were purchased from Genechem (Shanghai, China); The primers of LATS1, YAP, CTGF, E-cadherin, Ki-67 and MMP-2 were synthesized by ABI (Framingham, MA, USA). The antibodies of LATS1, YAP, CTGF and E-cadherin were purchased from Cell Signaling Technologies (Boston, MA, USA).

### Reagents

17 AAG was obtained from Invitrogen (Carlsbad, CA, USA). DMEM and foetal bovine serum (FBS) were from Thermo Fisher Scientific Inc (Waltham, MA, USA); TRIzol Reagent and Lipofectamine 2000 were from Invitrogen; M-MLV Reverse Transcriptase was from Promega (Madison, WI, USA); SYBR Green Master Mixture was from Takara (Otsu, Japan). ECL-PLUS/Kit was from GE Healthcare (Piscataway, NJ, USA).

### Clinical samples and data

Tissue microarray was prepared for IHC test. LAC and the corresponding ANCT were obtained from biopsy in a total of 75 consecutive cases admitted in our hospital from January 2009 to December 2012. The baseline characteristics of the patients before neo-adjuvant chemotherapy were summarized in Table S1. The study was approved by Medical Ethics Committee of Shanghai Jiao Tong University and written informed consent was obtained from the patients or their parents before sample collection. Two pathologists, respectively, reviewed all of the cases.

### Tissue microarray

The advanced tissue arrayer (ATA-100, Chemicon International, Tamecula, CA, USA) was used to create holes in a recipient paraffin block and to acquire cylindrical core tissue biopsies with a diameter of 1 mm from the specific areas of the ‘donor’ block. The tissue core biopsies were transferred to the recipient paraffin block at defined array positions. The tissue microarrays contained tissue samples from 75 formalin-fixed paraffin-embedded cancer specimens with known diagnosis, and corresponding ANCT from these patients. The block was incubated in an oven at 45°C for 20 min. to allow complete embedding of the grafted tissue cylinders in the paraffin of the recipient block, and then stored at 4°C until microtome sectioning.

### Immunohistochemical staining

LATS1, E-cadherin and YAP antibodies were used for IHC detection of protein expression in LAC tissues. LATS1, E-cadherin and YAP antibodies were used at 1:100 dilutions. Endogenous peroxidase was inhibited by incubation with freshly prepared 3% hydrogen peroxide with 0.1% sodium azide. Non-specific staining was blocked with 0.5% casein and 5% normal serum. Tissue microarrays were incubated with biotinylated antibodies and horseradish peroxidase. Staining was developed with diaminobenzidine substrate and sections were counterstained with haematoxylin. PBS replaced LATS1, E-cadherin and YAP antibodies in NCs. The expression of LATS1 was semi-quantitatively estimated as the total immunostaining scores, which were calculated as the product of a proportion score and an intensity score. The proportion and intensity of the staining was evaluated independently by two observers. The proportion score reflected the fraction of positive staining cells (0, none; 1, ≤10%; 2, 10% to ≥25%; 3, *>*25% to 50%; 4, >50%), and the intensity score represented the staining intensity (0, no staining; 1, weak; 2, intermediate; 3, strong). Finally, a total expression score was given ranging from 0 to 12. Based on the analysis in advance, LATS1 expression was categorized into low (score 0–3) and high (score 4–12). The scoring was independently assessed by two pathologists.

### Cell culture and pre-treatment with 17-AAG

Lung adenocarcinoma cells (A549 and LETPα-2) were cultured in DMEM medium supplemented with 10% heat-inactivated FBS, 100 U/ml of penicillin and 100 μg/ml of streptomycin. They were all placed in a humidified atmosphere containing 5% CO_2_ at 37°C. LAC cells were pre-treated with different concentrations of 17-AAG (0, 0.3, 1.0, 3 μmol/l). Cells were subcultured at a 1:5 dilution in 300 μg/ml G418-containing medium, and the cells treated by Lv-LATS1, Lv-LAST1-siRNA or 17-AAG were selected and expanded for further study. The Lv-LATS1/Lv-LAST1-siRNA virus vector-infected clone, the NC vector-infected cells, and LAC cells were named as Lv-LATS1/Lv-LAST1-siRNA, NC and CON groups, respectively.

### Quantitative real-time PCR

To quantitatively determine the mRNA expression levels of LATS1, YAP, CTGF, E-cadherin, Ki-67 and MMP-2 in LAC cells, real-time PCR was used. Total RNA of each clone was extracted with TRIzol according to the manufacturer's protocol. Reverse-transcription was carried out using M-MLV and cDNA amplification was carried out using SYBR Green Master Mix kit according to the manufacturer's protocol. Target genes were amplified using specific oligonucleotide primer and human glyceraldehyde-3-phosphate dehydrogenase (GAPDH) gene was used as an endogenous control. The PCR primer sequences were as follows: LATS1, 5′-CAGGTGGTCCTTTCAGAG-3′ and 5′-GGCACAGAGGTCCATAAC-3′; YAP, 5′-GTGGCACCTATCACTCTC-3′ and 5′-GTCTGGGAAACGGTTCTG-3′; CTGF, 5′-AAGGTGTGGCTTTAGGAG-3′ and 5′-TTGATGGCTGGAGAATGC-3′; E-cadherin, 5′-TGCTCTTGCTGTTTCTTCGG-3′ and 5′-TGCCCCATTCGTTCAAGTAG-3′; Ki-67, 5′-GGGTTACCTGGTCTTAGTTC-3′ and 5′-GTTGAGGCTGTTCCTTGATG-3′; MMP-2, 5′-CGGTGCCCAAGAATAGATG-3′ and 5′-AGGAGAAGAGCCTGAAGTG-3′; GAPDH, 5′-CAACGAATTTGGCTACAGCA-3′ and 5′-AGGGGTCTACATGGCAACTG-3′. Data were analysed using the comparative Ct method (2^−ΔΔCt^). Three separate experiments were performed for each clone.

### Western blot assay

Lung adenocarcinoma cells were harvested and extracted using lysis buffer (Tris-HCl, SDS, Mercaptoethanol, Glycerol). Cell extracts were boiled for 5 min. in loading buffer and then equal amount of cell extracts were separated on 15% SDS-PAGE gels. Separated protein bands were transferred into polyvinylidene fluoride membranes and the membranes were blocked in 5% skim milk powder. The primary antibodies against p-LATS1, YAP, CTGF and E-cadherin were diluted according to the instructions of antibodies and incubated overnight at 4°C. Then, horseradish peroxidase-linked secondary antibodies were added at a dilution ratio of 1:1000, and incubated at room temperature for 2 hrs. The membranes were washed with PBS for three times and the immunoreactive bands were visualized using ECL-PLUS/Kit according to the kit's instruction. The relative protein level in different groups was normalized to GAPDH concentration. Three separate experiments were performed for each clone.

### Cell proliferation assay

Cell proliferation was analysed with the MTT assay. Briefly, cells pre-treated with 17-AAG were incubated in 96-well-plates at a density of 1 × 10^5^ cells per well with DEME medium supplemented with 10% FBS. Cells were treated with 20 μl MTT dye at 48 hrs, and then incubated with 150 μl of DMSO for 5 min. The colour reaction was measured at 570 nm with enzyme immunoassay analyser (Bio-Rad, USA). The proliferation activity was calculated for each clone.

### Wound-healing assay

Lung adenocarcinoma cells were plated in each well of a 6-well culture plate and allowed to grow to 90% confluence. Treatment with 17-AAG was then performed. On the next day, a wound was created using a micropipette tip. The migration of cells towards the wound was monitored daily, and images were captured at time intervals of 24 hrs. Each assay was repeated three times.

### Transwell invasion assay

Transwell filters were coated with matrigel (3.9 μg/μl, 60–80 μl) on the upper surface of a polycarbonic membrane (diameter 6.5 mm, pore size 8 μm). After incubating at 37°C for 30 min., the matrigel solidified and served as the extracellular matrix for analysis of tumour cell invasion. Harvested cells (1 × 10^5^) in 100 μl of serum free DMEM were added into the upper compartment of the chamber. A total of 200 μl conditioned medium derived from NIH3T3 cells was used as a source of chemoattractant, and was placed in the bottom compartment of the chamber. After 24 hrs incubation at 37°C with 5% CO_2_, the medium was removed from the upper chamber. The non-invaded cells on the upper side of the chamber were scraped off with a cotton swab. The cells that had migrated from the matrigel into the pores of the inserted filter were fixed with 100% methanol, stained with Haematoxylin, and mounted and dried at 80°C for 30 min. The number of cells invading through the matrigel was counted in three randomly selected visual fields from the central and peripheral portion of the filter using an inverted microscope (200× magnification). Each assay was repeated three times.

### Cell apoptosis analysis

To detect cell apoptosis, LAC cells were trypsinized, washed with cold PBS and resuspended in binding buffer according to the instruction of the apoptosis kit. FITC-AnnexinV and PI were added to the fixed cells for 20 min. in darkness at room temperature. Then, Annexin V binding buffer was added to the mixture before the fluorescence was measured on FAC sort flow cytometer. The cell apoptosis was analysed using the Cell Quest software (Becton Dickinson, Mountain View, CA, USA). Three separate experiments were performed for each clone.

### Cell cycle analysis

To detect cell cycle variation, LAC cells were trypsinized, washed by PBS and fixed with 80% cold ethanol overnight at −20°C. After PBS washing, the fixed cells were stained with PI in the presence of RNase A for 30 min. at room temperature in darkness. Each sample was filtered through a 50 μm nylon filter to obtain single-cell suspension. The samples were then analysed on FACsort flow cytometer (Becton Dickinson). ModFit3.0 software (Verity Software House, Topsham, ME, USA) was used for cell cycle analysis. Three separate experiments were performed for each clone.

### Subcutaneous tumour model and drug intervention

Six-week-old female immune-deficient nude mice (BALB/c-nu) were bred at the laboratory animal facility (Institute of Chinese Academy of Sciences, Shanghai, China), and were housed individually in microisolator ventilated cages with free access to water and food. All experimental procedures were performed according to the regulations and internal biosafety and bioethics guidelines of Shanghai Municipal Science and Technology Commission. Mice were injected subcutaneously with 1 × 10^7^ LAC cells in 50 μl of PBS pre-mixed with an equal volume of matrigel matrix (Becton Dickinson). Mice were monitored daily and developed a subcutaneous tumour. When the tumour size reached approximately 5 mm in length, they were surgically removed, cut into 1–2 mm^3^ pieces, and re-seeded individually into other ones, which were randomly assigned as untreated group and 17-AAG-treated groups. 17-AAG with 50 or 100 mg/kg was injected into subcutaneous tumours using a multi-site injection format. The tumour volume was measured with a caliper, using the formula volume + (length × width)^2^/2.

### Statistical analysis

SPSS 20.0 was used for statistical analysis. Kruskal–Wallis H-test and chi-squared test were used to analyse the expression rates in all groups. One-way anova was used to analyse the differences between groups. The LSD method of multiple comparisons was used when the probability for anova was statistically significant. Statistical significance was set at *P* < 0.05.

## Results

### The correlation of LATS1 and YAP expression with clinicopathological characteristics

**Fig 1:**
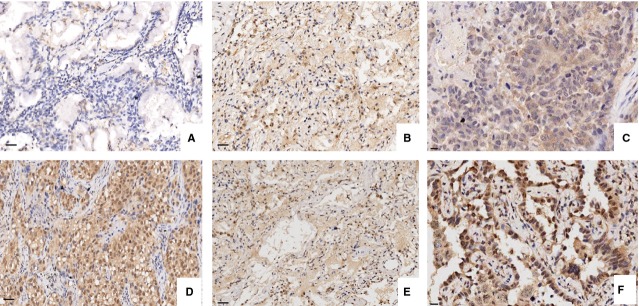
The expression of LATS1 in LAC examined by IHC staining (×200 and ×400). (A) Low expression of LATS1 in LAC tissues. (B) High expression of LATS1 in ANCT (adjacent non-cancerous tissues). (C) The positive expression of LATS1 was localized in the cytoplasm in LAC. (D) High expression of YAP in LAC tissues. (E) Low expression of YAP in ANCT. (F) YAP was localized in the nucleus in LAC.

The expression of LATS1 and YAP proteins was evaluated by IHC staining. As shown in Figure1 and Table1, the positive staining of LATS1was found increased in ANCT but decreased in LAC tissues (Fig.1A and B; *P* < 0.001), whereas that of YAP was found highly expressed in LAC tissues compared with the ANCT (Fig.1D and E; *P* + 0.03). Spearman correlation analysis revealed the negative correlation of LATS1 with YAP expression (*r* + 0.432, *P* < 0.001). Form the cellular localization, LATS1 protein was mainly localized in the cytoplasm (Fig.1C), while YAP was in the nucleus (Fig.1F) in LAC cells. The association between LATS1 and YAP expression and various clinicopathological factors was analysed in Table2. The expression of LATS1 and YAP was correlated with lymphatic invasion of LAC (*P* + 0.015, *P* + 0.003), but they did not link to other factors including age, gender, tumour size, pathological stage and TNM stage (each *P* > 0.05).

**Table 1:** Expression of LATS1 and YAP proteins in LAC tissues

Target	Group	Total	*N*				Positive rate (%)	χ^2^	*P*
			−	+	++	+++			
LATS1	LAC	75	55	12	5	3	26.7	21.353	<0.001
	ANCT	75	24	35	11	5	68.0		
YAP	LAC	75	18	30	16	11	76.0	4.722	0.03
	ANCT	75	33	21	14	7	56.0		

LAC, lung adenocarcinoma; ANCT, adjacent non-cancerous tissues.

**Table 2:** The correlation of LATS1 and YAP expression with clinicopathological characteristics of LAC patients

Variables	Cases (*n*)	LATS1		*P*	YAP		*P*
		−	+		−	+	
Total	75	55	20		18	57	
Age (years)							
≥60	40	32	8	0.166	7	33	0.162
<60	35	23	12		11	24	
Gender							
Male	40	31	9	0.386	13	27	0.067
Female	35	24	11		5	30	
Tumour size (cm)							
≥5	44	33	11	0.699	12	32	0.432
<5	31	22	9		6	25	
Pathological stage							
I + II	62	45	17	0.749	13	49	0.182
III	13	10	3		5	8	
TNM stage							
T1 + T2	62	47	15	0.293	15	47	0.932
T3	13	8	5		3	10	
Lymph node metastasis							
Negative	35	21	14	0.015	14	21	0.003
Positive	40	34	6		4	36	

### The effects of 17-AAG on the expression of LATS1, p-LATS1, YAP, CTGF and E-cadherin

**Fig 2:**
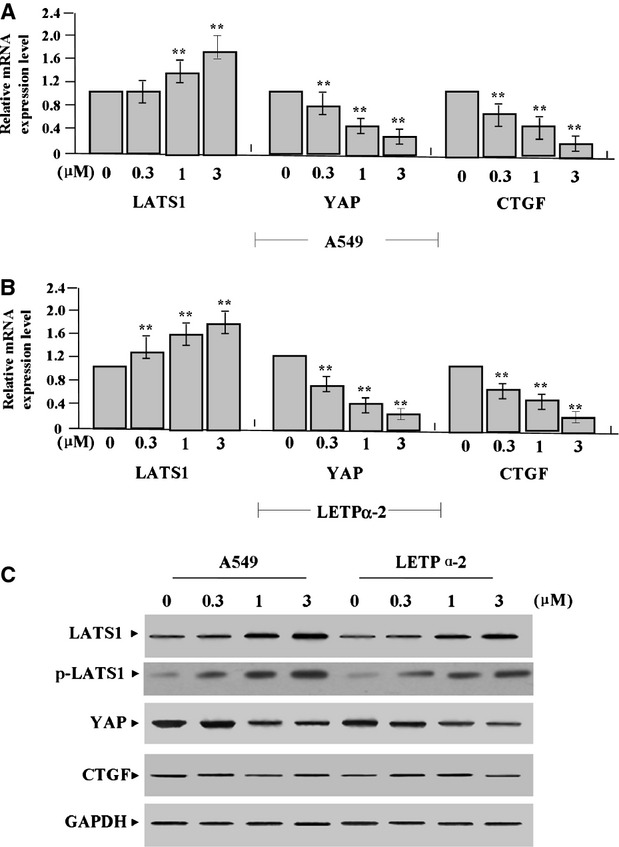
Effects of 17-AAG on the expression of LATS1, p-LATS1, YAP and CTGF. (A and B) The mRNA expression levels of YAP and CTGF, indicated by Real-time PCR assay, were markedly decreased, while those of LATS1 and p-LATS1 were increased in 17-AAG treated groups for 24 hrs compared to the untreated group (each **P < 0.01). (C) The expression levels of YAP and CTGF proteins, indicated by Western blot assay, were down-regulated, while LATS1 was upregulated in a dose-dependent manner in 17-AAG treated groups compared to the untreated group.

**Fig 3:**
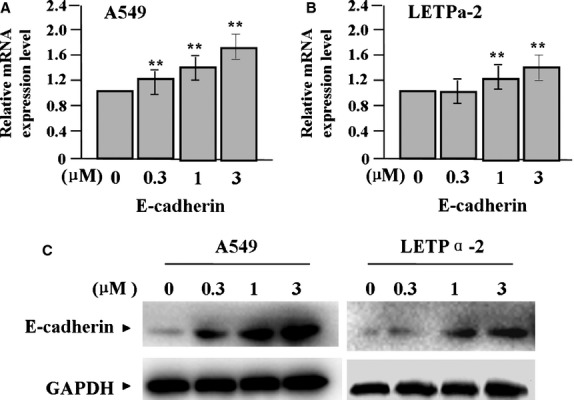
Effect of 17-AAG on the expression of E-cadherin. The expression of E-cadherin mRNA and protein, indicated by real-time PCR (A and B) and Western blot assay (C), was upregulated in 17-AAG treated groups in a dose-dependent manner compared to the untreated group (each **P < 0.01).

Huntoon *et al*. have reported that LATS1/2 may be the HSP90 clients in LAC cell, and that 17-AAG leads to decreased LATS1/2 level and increased CTGF level 21. To confirm the effects of 17-AAG on the expression of LATS1, p-LATS1, YAP and CTGF, we examined their expression levels in LAC cells (A549 and LETPα-2) treated with 17-AAG for 24 hrs by Real-time PCR (Fig.2A and B) and Western blot assays (Fig.2C), indicating that the expression of YAP and CTGF was markedly decreased, while that of LATS1 and p-LATS1 was increased in a dose-dependent manner in 17-AAG treated groups compared to untreated group (each **P < 0.01). Then, we further explored the effect of 17-AAG on the expression of E-cadherin in LAC cells, and found that E-cadherin expression was upregulated in 17-AAG treated groups in a dose-dependent manner compared to untreated group (each **P < 0.01, Fig.3A–C).

### The effects of 17-AAG on cell proliferation

**Fig 4:**
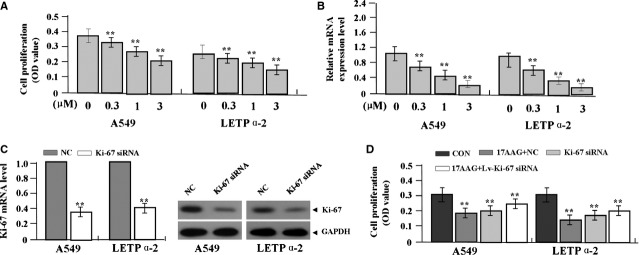
Effects of 17-AAG on cell proliferation. (A) 17-AAG diminished cell proliferative activities of LAC cells (A549 and LETPα-2) in a dose-dependent manner compared to the untreated group (each **P < 0.01). (B) The amount of Ki-67 mRNA, indicated by real-time PCR assay, was significantly reduced in a dose-dependent manner in 17-AAG treated groups compared to the untreated group (**P < 0.01). (C) After Lv-Ki-67-siRNA was transfected into LAC cells for 24 hrs, the expression level of Ki-67 mRNA and protein was markedly knocked down. (D) Cell proliferation was significantly repressed in 17-AAG+NC group compared to the 17-AAG+Lv-Ki-67-siRNA, Lv-Ki-67-siRNA and CON groups (**P < 0.01).

To define the effects of 17-AAG on cell growth, we examined proliferative activities of LAC cells (A549 and LETPα-2) by MTT assay. We found that 17-AAG significantly diminished the proliferative activities of LAC cells in a dose-dependent manner compared to untreated group (each **P < 0.01; Fig.4A). To verify the effect of 17-AAG on endogenous expression of Ki-67 through transcriptional repression, Ki-67 expression was examined by Real-time PCR, indicating that the amount of Ki-67 mRNA was significantly lessened in a dose-dependent manner in 17-AAG treated groups compared to the untreated group (**P < 0.01; Fig.4B). Then, Lv-Ki-67-siRNA was transfected into LAC cells, and Ki-67 could be verified to be knocked down in LAC cells (Fig.3C). Lv-Ki-67-siRNA was further transfected 17-AAG (1.0 μmol/l) treated-LAC cells, indicating that 17-AAG had better inhibitory effects on LAC proliferation in NC-transfected cells than Lv-Ki-67-siRNA transfected cells (Fig.4D), suggesting that 17-AAG might inhibit growth of LAC cells through down-regulation of Ki-67 expression.

### The effects of 17-AAG on cell migration and invasion

**Fig 5:**
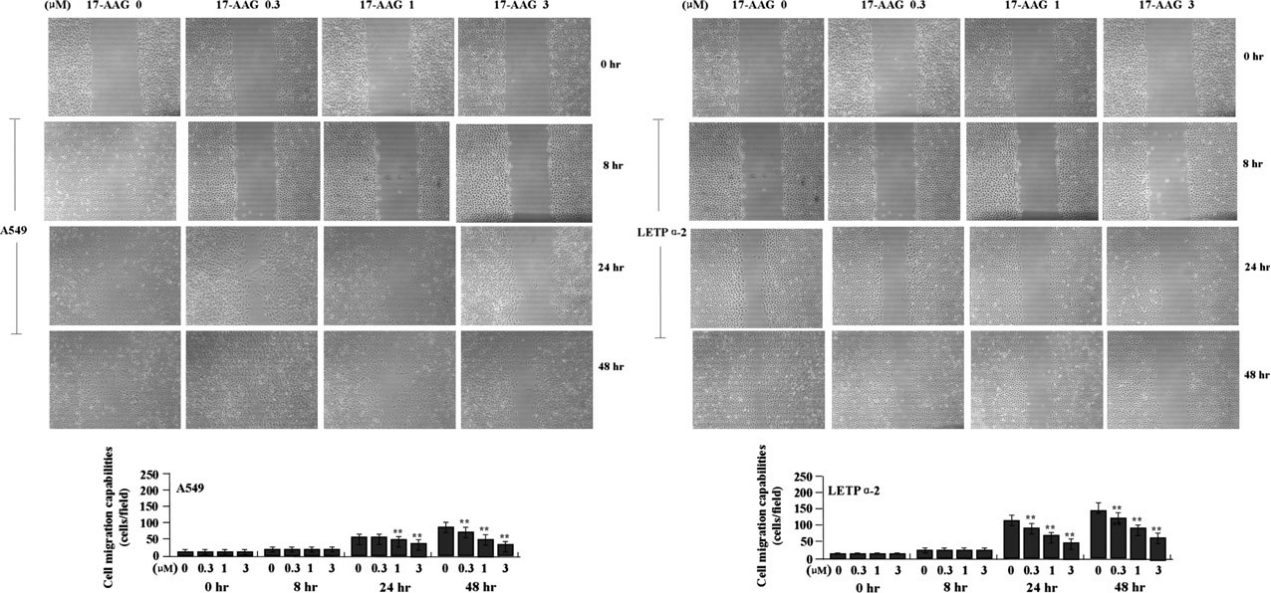
Effects of 17-AAG on cell migration. After the LAC cells were treated with 17-AAG for 24 hrs, wound-healing assay showed that cell migrating capabilities were reduced in 17-AAG treated groups compared to the untreated group in a dose-dependent manner (**P < 0.01).

**Fig 6:**
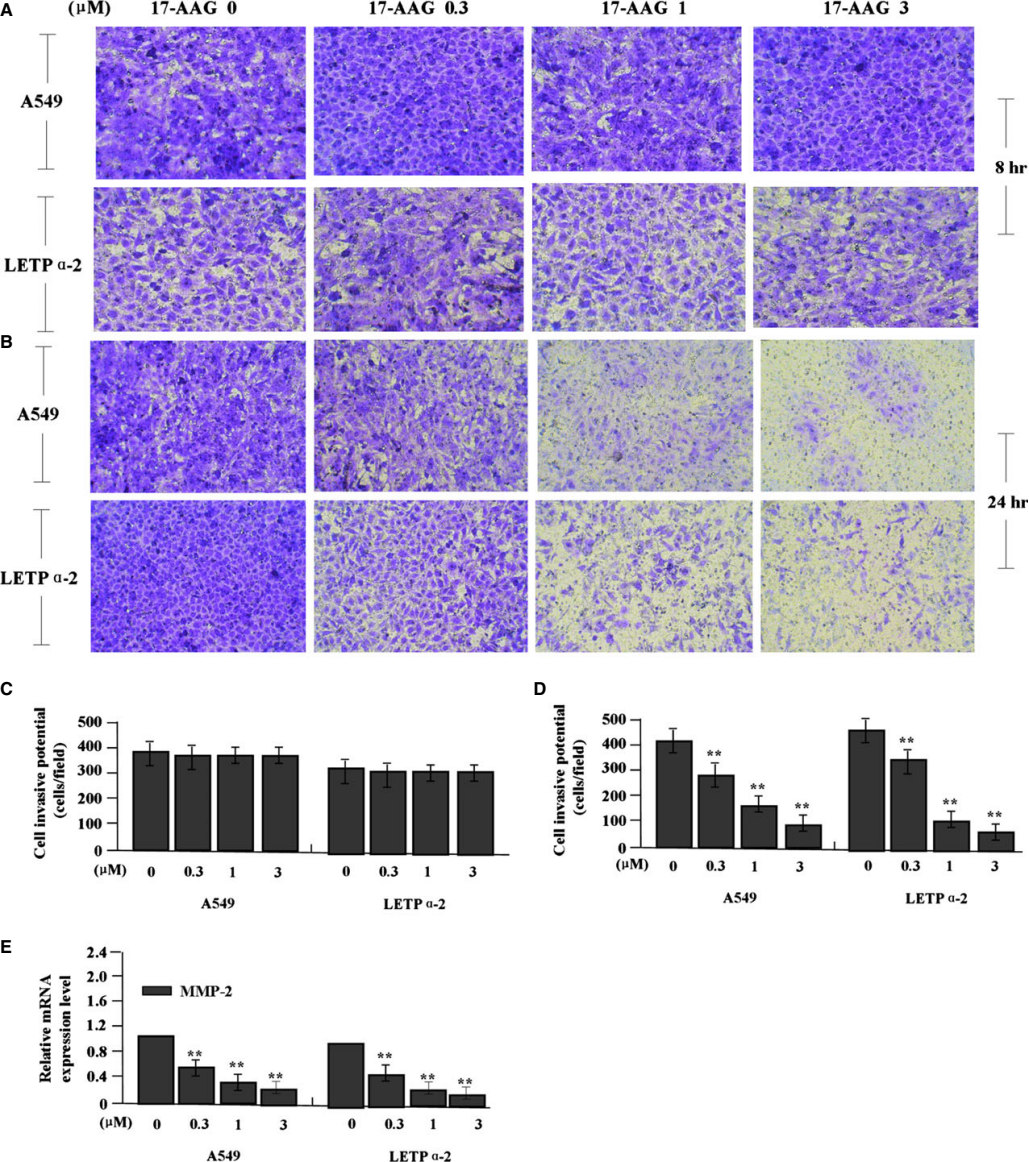
Effects of 17-AAG on cell invasion. (A and C) After LAC cells were treated with 17-AAG for 8 hrs, Transwell assay showed that cell invasive potential had no significant difference between the treatment groups (*P* > 0.05). (B and D) After 24 hrs, the invasive potential of LAC cells, indicated by Transwell assay, was significantly weakened in 17-AAG treated groups compared to the untreated group in a dose-dependent manner (**P < 0.01). (E) The amount of MMP-2 mRNA, indicated by real-time PCR was significantly decreased in a dose-dependent manner in 17-AAG treated groups compared to the untreated group (**P < 0.01).

To determine the effect of 17-AAG on migration and invasion of LAC cells (A549 and LETPα-2), Wound-healing and Transwell assays were performed. After LAC cells were treated with 17-AAG for 8 hrs, the migrating capabilities had no significant difference between the 17-AAG-treated groups (*P* > 0.05), but after 24 hrs the migrating capabilities of LAC cells in 17-AAG treated groups turned significantly slower than those in untreated group (Fig.5, **P < 0.01). The invasive potential was determined on the basis of the ability of cells to invade a matrix barrier containing laminin and type IV collagen, the major components of the basement membrane. Representative micrographs of Transwell filters were shown in Figure6.

After LAC cells were treated with 17-AAG for 8 hrs, the invasive potential had no significant difference between the 17-AAG-treated groups (Fig.6A, C, *P* > 0.05), but after 24 hrs the invasive potential of LAC cells was significantly weakened in 17-AAG treated groups compared to the untreated group in a dose-dependent manner (Fig.6B and D, **P < 0.01). The endogenous expression of MMP-2, indicated by Real-time PCR (Fig.6E) was down-regulated in 17-AAG treated groups compared to the untreated group (**P < 0.01), indicating that 17-AAG might inhibit migration and invasion of LAC cells through down-regulation of MMP-2 expression.

### The effects of 17-AAG on cell apoptosis and cycle distribution

**Fig 7:**
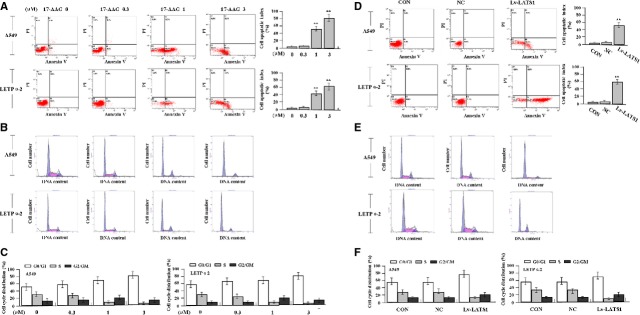
Effects of 17-AAG on cell apoptosis and cycle distribution. (A) The apoptotic indexes of LAC cells were remarkably higher in 17-AAG treated groups than the untreated group (**P < 0.01). (B and C) Cell cycle kinetics showed that, the G_0_/G_1_ phase fraction was increased, while S phase fraction was decreased, suggesting that cell cycle was arrested in G_0_/G_1_ phase in 17-AAG treated groups compared to the untreated group. (D) Cell apoptotic indexes were much higher in Lv-LATS1 group than the CON and NC groups (**P < 0.01). (E and F) The G_0_/G_1_ phase fraction was increased, while S phase fraction was decreased in Lv-LATS1 group compared to the CON and NC groups, suggesting that LATS1 could induce cycle arrest in G_0_/G_1_ phase.

To assess the effect of 17-AAG on apoptosis and cycle distribution of LAC cells, flow cytometric analysis was carried out. After LAC cells were treated with 17-AAG for 24 hrs, apoptotic indexes of LAC cells in 17-AAG treated groups were remarkably higher than the untreated group (**P < 0.01, Fig.7A). Cell cycle kinetics showed that, the G_0_/G_1_ phase fraction was increased, while S phase fraction was decreased, suggesting that cell cycle was arrested in G_0_/G_1_ phase in 17-AAG treated groups compared to the untreated group (Fig.7B and C). Then, we investigated the effects of LAST1 overexpression on the cell apoptosis and cycle distribution in LAC cells. After LAC cells were transfected with Lv-LATS1 for 24 hrs, we found that cell apoptotic indexes were much higher than the control (CON) and NC groups (**P < 0.01, Fig.7D). Cell cycle distribution indicated that the G_0_/G_1_ phase fraction was increased, while S phase fraction was decreased in Lv-LATS1 group compared to the CON and NC groups (Fig.7E and F), suggesting that LATS1 could induce cell apoptosis and cycle arrest in G_0_/G_1_ phase.

### 17-AAG inhibition of LAC proliferation *via* LATS1/YAP signalling

**Fig 8:**
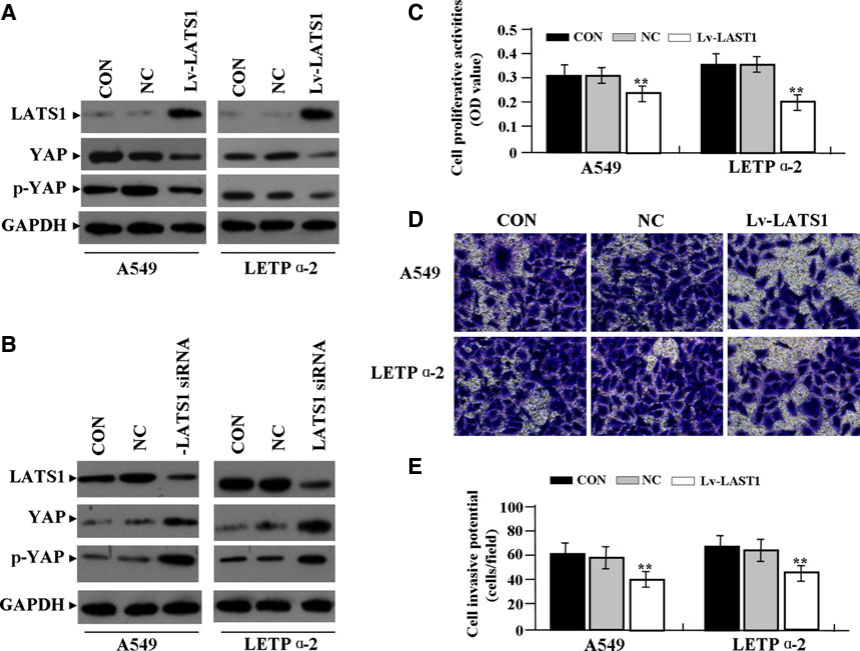
17-AAG inhibition of cell proliferation and invasion dependent of LATS1/YAP signalling. (A and B) Ectopic expression of LATS1 and siRNA-dependent silencing of LATS1 were confirmed by western blotting. The downstream regulators YAP and p-YAP were found down-regulated in LATS1-overexpressing cells but upregulated in LATS1-silenced cells. Cell proliferative activity (C) and invasive potential (D and E) of LAC cells were apparently decreased in Lv-LATS1 group compared to NC and CON groups (**P < 0.01).

**Fig 9:**
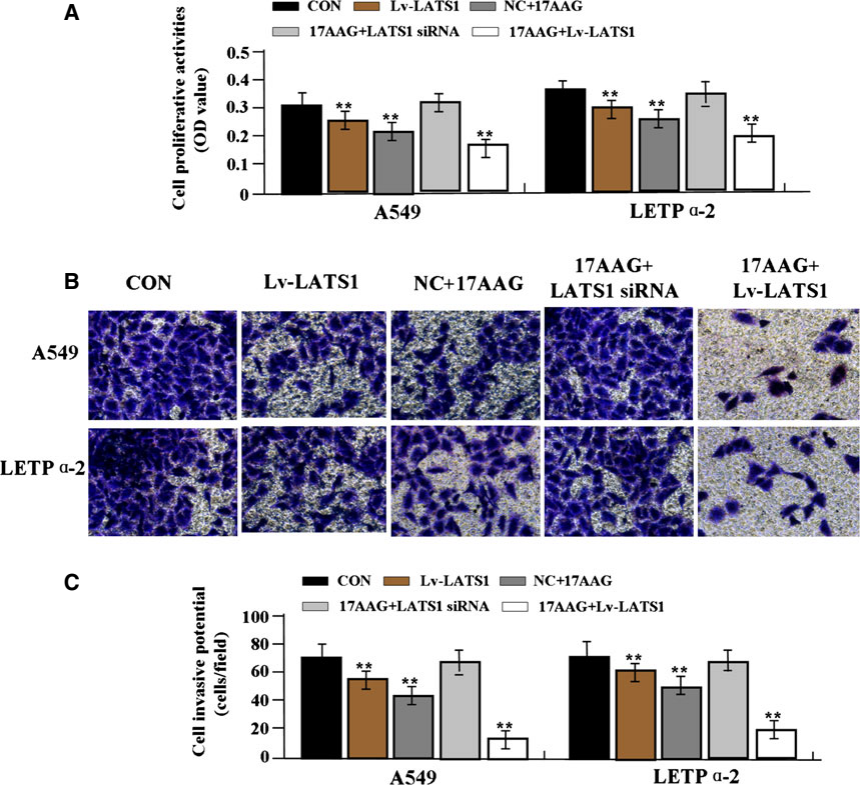
The effects of LATS1 on 17-AAG-treated LAC cells. Lv-LATS1 or Lv-LATS1-siRNA was transfected into 17-AAG (1.0 μmol/l) treated-LAC cells, indicating that cell proliferation (A) and invasion (B and C) of LAC cells were significantly weakened in 17-AAG+Lv-LATS1 group compared to the 17-AAG and CON groups (**P < 0.01), but 17-AAG had no inhibitory effect in Lv-LATS1-siRNA transfected cells compared to non-transfected cells.

To determine whether LATS1/YAP signalling mediated the inhibitory effects of 17-AAG on LAC proliferation, we examined the activation status of the LATS1/YAP pathway by western blotting, and assessed cell proliferation and invasion by MTT and Transwell assays. First, ectopic expression of LATS1 and siRNA-dependent silencing of LATS1 were confirmed in LAC cells by western blotting (Fig.8A and B). The downstream regulators YAP and p-YAP were found down-regulated in LATS1-overexpressing cells but upregulated in LATS1-silenced cells (Fig.8A and B). Then, after Lv-LATS1 was transfected into LAC cells for 24 hrs, we found that cell proliferative activity (Fig.8C) and invasive potential (Fig.8D and E) of LAC cells were apparently decreased in Lv-LATS1 group compared to NC and CON groups (**P < 0.01). Lv-LATS1 and Lv-LATS1-siRNA were, respectively, transfected into 17-AAG (1.0 μmol/l) treated-LAC cells, our results showed that cell proliferative activity and invasive potential of LAC cells were significantly weakened in 17-AAG+Lv-LATS1 group compared to the 17-AAG and CON groups (**P < 0.01), but 17-AAG had no inhibitory effects on cell biological behaviour in Lv-LATS1-siRNA transfected cells compared to non-transfected cells (Fig.9A–C).

### The effect of 17-AAG on xenograft tumour growth

**Fig 10:**
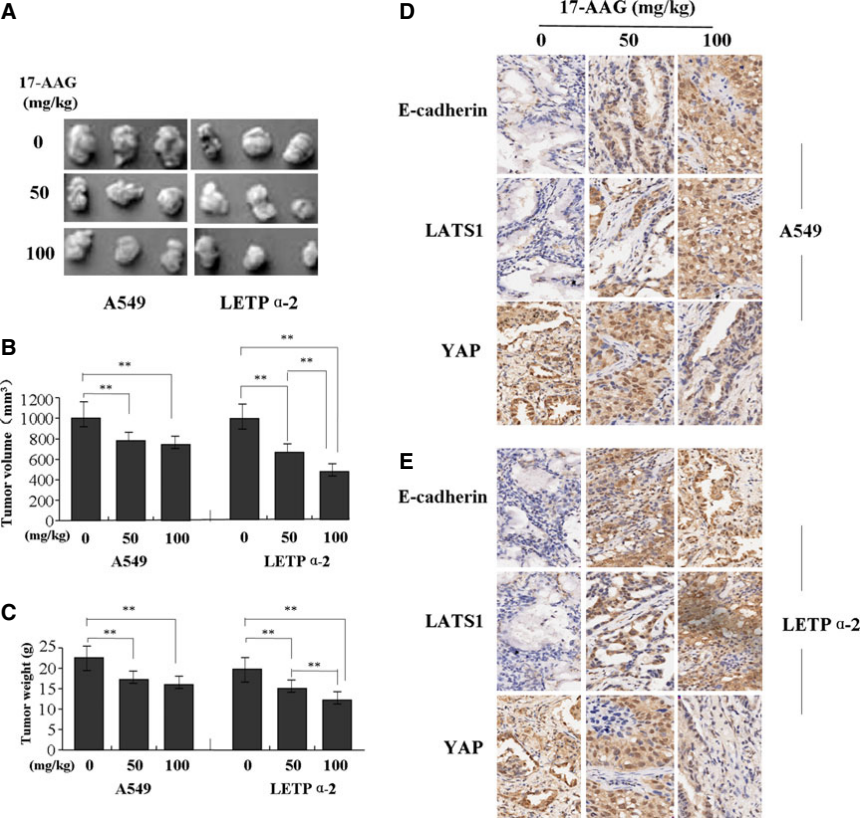
Effects of 17-AAG on LAC xenograft tumour growth. (A and B) A549 and LETPα-2 xenograft tumours treated with 17-AAG became substantially smaller than those of untreated group. (C) The average weight and volume of the tumours in 17-AAG treated groups were significantly smaller than those of the untreated group (each *P* < 0.01). (D and E) Immunohistochemical analysis of E-cadherin, LATS1 and YAP expression in cancer sections from mice treated with 17-AAG. Increased expression of E-cadherin and LATS1 but decreased expression of YAP were observed in the 17-AAG-treated groups compared to untreated group.

Our *in vitro* experiments demonstrated inhibitory effects of 17-AAG on growth of LAC cells. Thus, it is necessary to further investigate the effect of 17-AAG on A549 and LETPα-2 xenograft tumour growth *in vivo*. During the whole tumour growth period, the tumour growth activity was measured. The tumours treated with 17-AAG turned substantially smaller in comparison with the untreated group (Fig.10A). When the tumours were harvested on 18th day, the average weight and volume of the tumours in 17-AAG-treated groups were significantly smaller than those of the untreated group (Fig.10B and C). Then, the tumour samples were removed for immunohistochemical analysis of E-cadherin, LATS1 and YAP expression. Representative sections from mice treated with 17-AAG could be seen in Figure10D and E. Increased expression of E-cadherin and LATS1 but decreased expression of YAP were observed in the 17-AAG-treated groups compared to untreated group.

## Discussion

Lung cancer was the most commonly diagnosed cancer and leading cause of cancer death in males worldwide in 2008. Among females, it was the fourth most commonly diagnosed cancer and the second leading cause of cancer death 22. Current best approach for the treatment of cancer is complete surgical removal of the tumour and adjacent lymph nodes. However, the efficacy of this therapeutic approach alongside hormone, radiotherapy and chemotherapy is very limited 23. Therefore it is urgently needed to explore the effective targets and therapeutic drugs for the treatment of cancer.

LATS1/YAP pathway is one major conserved mechanism governing cell contact inhibition, organ size control and cancer development 13. The decreased expression of LATS1 is inversely correlated with lymph node metastasis in gastric cancer, and one of the reasons for loss of LATS1 expression lies in the hypermethylation of LATS1 in the promoter regions 24. Low expression of LATS1 is associated with poor prognosis and contributes to the biologically aggressive phenotype in breast cancer 25. However, few studies show that LATS1 is overexpressed in cervical cancers and basal-like breast cancers 26, and the LATS1 methylation statuses do not correlate with survival of lung cancer patients 27. In the present study, we found that the expression of LATS1 was down-regulated in the cytoplasm but YAP was upregulated in the nucleus in LAC tissue cells compared to the ANCT, suggesting that the tumourigenesis of LAC might be associated with the decrease of cytoplasmic accumulation of LATS1 and the increase of nuclear accumulation of YAP. Moreover, the expression of LATS1 was inversely correlated with the YAP expression and lymphatic invasion of the tumour, indicating that LATS1/YAP signalling may participate in the progression of LAC.

Increasing evidence demonstrates the antitumour effects of 17-AAG in a variety of human malignancies, including suppression of cell growth and induction of apoptosis and cycle arrest in cholangiocarcinoma cells by inhibition of HSP90 function 28. In addition to regulating HSP90 signalling, 17-AAG leads to depletion of oncogenic proteins involved in multiple pathways, of which NF-κB and MAPK pathways can be activated in 17-AAG-resistant breast cancer cells 29. Suppression of heat shock factor-1 or hypoxia inducible factor pathway by 17-AAG impairs renal cancer cell growth, motility and angiogenesis 30,31. Interestingly, Huntoon *et al*. report that the HSP90 inhibitor 17-AAG disrupts LATS tumour suppressor pathway with decreased phosphorylation of the LATS substrate YAP involved in cell and tissue growth, and with increased CTGF implicated in tumour proliferation, metastasis and angiogenesis in human cancer cells 21. Similar with the study of Huntoon, we also found 17-AAG decreased the phosphorylation of YAP. But, different from that, our finding revealed that 17-AAG inhibited cell growth and invasion, and induced apoptosis and cycle arrest in LAC cells together with increased transcriptional level and phosphorylation of the LATS1, and down-regulation of CTGF expression *in vitro* and *in vivo*. The different reasons might be analysed as follows: on the one hand, our study showed that LATS1 functioned as a tumour suppressor in LAC cells and 17-AAG activated the LATS1 expression and then suppressed the phosphorylation of YAP, exerting the anti-LAC effects; On the other hand, we indirectly indicated that the LATS1 kinase might not be the *bona fide* HSP90 client and 17-AAG served as the activator of the LATS1 kinase in LAC cells. To confirm whether LATS1/YAP signalling mediated the inhibitory effects of 17-AAG on LAC cells, we investigated the function of LATS1 in LAC cells, indicating that ectopic expression of LATS1 repressed cell proliferation and invasion in LAC cells with decreased phosphorylation of YAP. Then, we demonstrated that 17-AAG inhibition of LAC proliferation and invasion depended on the activity of LATS1 in LAC cells, suggesting a novel molecular mechanism of 17-AAG in tumour therapy.

Epithelial-mesenchymal transition-related proteins including E-cadherin are known to be involved in the progression and metastasis of various cancers 32. E-cadherin expression is down-regulated in invasive LAC 33, controls cell proliferation and regulates the transduction of LATS1/YAP signalling 20 *via* modulating subcellular localization of the YAP 34 and transcriptional activation of CTGF 35. Our present study showed that 17-AAG upregulated the expression of E-cadherin and suppressed the development of LAC, suggesting that E-cadherin might mediate the regulation of 17-AAG on LATS1/YAP signalling in LAC cells. In addition to 17-AAG, other factors such as replication licensing factor (Cdc6) can negatively regulate the transcription activity of E-cadherin in human cancer 36, and no evidence indicates whether Cdc6 is implicated in the regulation of 17-AAG on E-cadherin. To our interest, secreted HSP90α down-regulates E-cadherin and enhances cancer cell migration and invasion 37, suggesting the effect of 17-AAG as a HSP90 inhibitor on E-cadherin expression.

Ki-67 is a nuclear protein that is expressed in proliferating cells, may be required for maintaining cell proliferation, and used as a marker for cancer cell proliferation. MMPs are important molecules involved in tumour metastasis. They are expressed on the tumour cell surface, activate pro-MMP to exacerbate the malignancy, and are considered a powerful indicator of distant metastasis of cancer 38. Our previous study has shown that LATS1/YAP signalling involves in tumour growth and invasion through regulation of the expression of Ki-67 and MMP-2 39. Furthermore, our present finding showed that, 17-AAG inhibited LAC cell growth and invasion with the decreased expression of Ki-67 and MMP-2, suggesting that LATS1/YAP signalling might mediate 17-AAG suppression of tumour growth and invasion *via* regulation of Ki-67 and MMP-2 expression in LAC cells.

In conclusion, our investigation reveal that decreased expression of LATS1 is related with the lymphatic invasion of human LAC, and 17-AAG as a HSP90 inhibitor suppresses growth and invasion of LAC cells *via* regulation of the LATS1/YAP signalling *in vitro* and *in vivo*, suggesting a promising therapeutic strategy for the treatment of LAC.

## Supporting Information

**Table S1** Clinicopathological data of LAC patients.
