# Supplementary material for: 17-AAG suppresses growth and invasion of lung adenocarcinoma cells via regulation of the LATS1/YAP pathway
Source: J Cell Mol Med. 2015 Feb 25;19(3):651–63. doi: 10.1111/jcmm.12469 (PMC4369821; doi:10.1111/jcmm.12469)
Supplement: Supplementary file 1 [file jcmm0019-0651-sd1.doc]

Table S1 Clinicopathologic data of LAC patients

| Variables | Number of cases (%) |
| --- | --- |
| Number of patients | 75 (100%) |
| *Age (years)* |  |
| ≤60 | 40 (53.3%) |
| ＞60 | 35 (46.7%) |
| *Sex* |  |
| Male | 40 (53.3%) |
| Female | 35 (46.7%) |
| *Tumor size (cm)* |  |
| ≤3. | 44 (58.7%) |
| ＞3 | 31 (41.3%) |
| *Pathological stage* |  |
| Ⅰ | 4 (5.4%) |
| Ⅱ | 58 (77.3%) |
| Ⅲ | 13 (17.3%) |
| *TNM stage* |  |
| T1 | 21 (28.0%) |
| T2 | 41 (54.7%) |
| T3 | 13 (17.3%) |
| *Lymphatic invasion* |  |
| Negative | 35 (46.7%) |
| Positive | 40 (53.3%) |
